# Early Systemic Immune Response to Silicone Breast Implants Analyzed by Flow Cytometry

**DOI:** 10.7759/cureus.78500

**Published:** 2025-02-04

**Authors:** Georgios E Papanikolaou, Dimitrios N Varvarousis, Georgios S Markopoulos, Konstantina Bouranta, Savvas Dimitriadis, Aikaterini Kitsouli, Theocharis Chatzoglou, Efstathios G Lykoudis

**Affiliations:** 1 Department of Plastic Surgery, University Hospital of Ioannina, Ioannina, GRC; 2 Medical School, University of Ioannina, Ioannina, GRC; 3 Department of Orthopaedics, University Hospital of Ioannina, Ioannina, GRC; 4 Haematology Laboratory, Unit of Molecular Biology and Translational Flow Cytometry, University Hospital of Ioannina, Ioannina, GRC

**Keywords:** flow cytometry, immune system, implant-based breast augmentation, lymphocytes, silicone breast implants

## Abstract

Background

Silicone breast implants are used in a variety of aesthetic and reconstructive procedures such as permanent implants or tissue expanders. As foreign biomaterials, they can potentially evocate either a local inflammatory reaction or a systemic immune response. This study aimed to quantify possible alterations in white blood cells and lymphocyte subpopulations in patients who underwent silicone implant-based breast augmentation through peripheral blood analysis with flow cytometry at three and 12 months postoperatively. The same patients acted as the control group with a preoperative peripheral blood collection.

Methodology

This retrospective study included 14 female patients (average age = 29 years; range = 18-44 years) who underwent breast augmentation with silicone implants. Flow cytometry was used to monitor their immunologic reactions. Peripheral blood samples were obtained before, three months, and 12 months after the operation. Flow cytometric analysis was performed to assess the distribution of total leukocytes and the expression of specific lymphocyte subsets using the following antibody panel: anti-CD3, anti-CD4, anti-CD8, anti-CD16/CD56, anti-CD19, and anti-CD25.

Results

The immunophenotypic analysis showed that the vast majority of the peripheral blood lymphocytes consisted of T lymphocytes (CD3+), followed by natural killer (NK) cells (CD3-/CD16+/CD56+, NK cells), and B lymphocytes (CD3-/CD19+) in all periods of the study. The peripheral blood CD3+ T-lymphocyte subsets were predominantly T-helper lymphocytes (CD3+/CD4+), followed by cytotoxic T lymphocytes (CTLs) (CD3+/CD8+, CTLs), and a small percentage were T-regulatory (Treg) cells (CD4+/CD25high, Treghigh cells). Interestingly, lymphocytes showed a slight increase and T-helper lymphocytes a slight decline postoperatively, with no evidence of statistically significant shifts. Overall, there was no statistically significant difference in the temporal distribution of the peripheral blood lymphocyte subpopulations.

Conclusions

To our knowledge, the novelty of our study is that the same patients who underwent breast augmentation with silicone implants were used as the control group. There was no evidence of an early specific systemic immune activation in these patients. Hence, flow cytometry could be a reliable method that can be used as an in vitro test to evaluate the biocompatibility of silicone implants and observe patients with immune activity for possible clinical manifestations of symptoms in the future.

## Introduction

Silicone-based biomaterials represent a broad class of synthetic polymers, which are used in the manufacturing of different types of medical devices and supplies [1,2]. Particularly, silicone implants are widely used in breast surgery, for both aesthetic augmentation and post-mastectomy prosthetic reconstruction [3-5]. Despite their popularity, the use of breast implants is associated with some adverse outcomes that can be divided into local complications and systemic effects, which can be attributed either to the initial surgery or to the implant itself [6-8]. However, as a material that is employed so widely must have the biologic capacity to adapt to the breast tissue in which it is inserted and meet all the necessary biocompatibility requisites.

Early host reactions following the implantation of silicone implants include acute and chronic inflammation, foreign body reaction, and the formation of a protective fibrous capsule as part of the natural healing process [9,10]. Furthermore, it is important to distinguish between this non-specific local inflammatory reaction and a possible systemic immune response and to clarify if there is a causal association between the two phenomena.

This study aimed to quantify the white blood cells and phenotype of the lymphocytes in the peripheral blood of patients with silicone breast implants using flow cytometric analysis and to investigate whether the early clinical course of these patients was associated with the activation of their systemic immune profile, represented by the circulating blood leukocyte subsets and lymphocyte subpopulations. Several CD markers were used as a reference to assess the leukocyte subtype profile. Given that current research mainly focuses on reporting the local complications of breast implants, we highlighted the importance of correlating the postoperative course with the systemic immune response of these patients after implant-based breast augmentation.

A version of this manuscript was previously presented as a meeting abstract at the 22nd Annual Meeting of the European Association of Plastic Surgeons (EURAPS) on June 3, 2011.

## Materials and methods

Study design

The study was conducted in accordance with the principles presented in the Declaration of Helsinki and was approved by the Scientific Committee of the University Hospital of Ioannina (protocol number: 02/07-01-2025, approval code: 01/14-01-2025 [θ.9], approval date: January 14, 2025). We retrospectively reviewed 14 female patients (average age = 29 years; range = 18-44 years) who underwent breast augmentation with permanent silicone implants for aesthetic and reconstructive purposes by the same plastic surgeon. Flow cytometry was used to monitor their immunologic reactions. Particularly, 12 patients underwent breast augmentation for cosmetic reasons and two patients underwent primary breast reconstruction due to Poland syndrome and tuberous breasts.

The surgical technique consisted of endoscopic placement of silicone implants in a subpectoral position and almost exclusively through an inframammary crease incision (13 out of 14 cases). In one case, the implant was placed through an anterior axillary approach. In all cases, microtextured (average surface roughness of 36.1 μm) and silicone gel-filled implants of high cohesiveness were used. In 12 patients, anatomic implants were placed, and in two patients, round-shaped implants were placed, with the size ranging from 215 cc to 350 cc.

Our study included healthy female patients, according to history and general physical examination, who did not previously have breast implants. The exclusion criteria included patients with a history or symptoms of connective tissue disease, rheumatologic disorder, autoimmune disease at the time of implantation, and history of malignancy; patients with other silicone devices implanted in their body; and patients undergoing immunosuppressant therapy. During the follow-up period, the clinical course and the presence of complications were recorded.

Flow cytometric analysis

Flow cytometric analysis parameters were selected based on established literature and standard immunophenotyping protocols. No additional markers were considered beyond those specified. Peripheral venous blood samples from patients were obtained preoperatively, as well as three and 12 months after the operation. The preoperative blood draw was performed to recruit the same patients as the control group. The three-month and 12-month timepoints were selected as at that time regular follow-up was conducted, and the healing process as well as tissue remodeling and implant settling occurred.

For the analysis, 5 mL of blood drawn by standard venipuncture was placed into tubes containing ethylenediaminetetraacetic acid as an anticoagulant and transported to the laboratory within 24 hours for testing. Subsequently, flow cytometric analysis was performed to assess the distribution of total leukocytes, particularly the expression of specific lymphocyte subsets, using monoclonal antibodies against CD3, CD4, CD8, CD16/CD56, CD19, CD25, and CD45 (specific clones include anti-CD3 Leu-4 SK7, anti-CD4 Leu-3a SK3, anti-CD8 Leu-2a SK1, anti-CD16+56 Leu-11c + Leu-19 B73.1 MY31, anti-CD19 Leu-12 4G7, anti-CD25 IL-2Rα 2A3, and anti-CD45 Hle-1 2D1).

Initially, 100 μL of blood sample was added to the appropriate tubes and mixed with 10 μL of mouse monoclonal antibodies directly conjugated with fluorescein isothiocyanate or phycoerythrin. The samples were then incubated at room temperature out of direct light for 15 minutes. After that, erythrocytes were lysed by incubation with 2 mL of FACSLyse solution (Becton Dickinson, San Jose, CA, USA). Following the lysis of erythrocytes, peripheral blood mononuclear cells (PBMCs) were washed with 2 mL of phosphate-buffered saline (PBS) solution and isolated with centrifugation at 3500 rpm for 10 minutes. Cells were then washed again adding 2 mL of PBS, and a second round of centrifugation was performed to eliminate any erythrocytic residual. The cell pellet was resuspended in 0.5 mL of PBS for fixation. Lymphocytes were gated by forward and right-angle light scattering, and flow cytometric acquisition was performed using FACSScan flow cytometer (Becton Dickinson, San Jose, CA, USA) and CellQuest V3.1 analysis software (Becton Dickinson, San Jose, CA, USA).

Statistical analysis

Data were analyzed using SPSS version 18.0 (SPSS Inc., Chicago, IL, USA). The Kolmogorov-Smirnov test and Shapiro-Wilk test of normality were used to determine whether the data were normally distributed. For data analysis, the repeated-measures analysis of variance test and the non-parametric Friedman test were used. Statistical comparisons between the two groups of patients were made using the t-test for normally distributed variables and the Mann-Whitney test for non-normally distributed data. The means and standard deviations of the variables were recorded for their descriptive statistical analysis. A p-value <0.05 was considered to represent a statistically significant difference at a confidence interval of 95%.

## Results

Clinical profile

The clinical and immunological outcomes observed in this study were largely unremarkable, indicating stable profiles across all evaluated parameters. Clinically, the postoperative course was uneventful in nearly all cases, except for one patient who developed minimal hypertrophic scarring at the incision site but without any other complaint. Nevertheless, the functional and aesthetic results were optimal for all patients throughout the follow-up period, including the one-year mark.

Flow cytometry analysis

Flow cytometric analysis was conducted on peripheral venous blood samples collected preoperatively, as well as at three and 12 months postoperatively. This analysis revealed no statistically significant differences in the distribution of peripheral white blood cells or lymphocyte subsets over time. The temporal distribution of polymorphonuclear cells, monocytes, and lymphocytes remained consistent, with mean percentages for these subsets showing only minor, non-significant fluctuations (Table 1). Specifically, lymphocytes accounted for 31.24% ± 4.89% preoperatively, increasing slightly to 33.26% ± 5.78% at three months and 33.31% ± 5.11% at 12 months postoperatively (p = 0.375). Similarly, the proportions of polymorphonuclear cells and monocytes showed stable trends, with no evidence of statistically significant shifts (p = 0.461 and p = 0.468, respectively).

**Table 1:** Peripheral white blood cell distribution in patients with silicone breast implants. SD: standard deviation

White blood cells	Mean value (%) ± SD			P-value
	Preoperatively	3 months postoperatively	12 months postoperatively	
Polymorphonuclear cells	60.36 ± 6.16	58.49 ± 6.91	58.16 ± 4.65	0.461
Monocytes	5.75 ± 1.64	5.19 ± 1.89	5.24 ± 1.51	0.468
Lymphocytes	31.24 ± 4.89	33.26 ± 5.78	33.31 ± 5.11	0.375

Further immunophenotypic analysis demonstrated that T lymphocytes (CD3+) consistently represented the majority of peripheral blood lymphocytes, followed by natural killer (NK) cells (CD3-/CD16+/CD56+, NK cells) and B lymphocytes (CD3-/CD19+) (Table 2). At all three time points, T lymphocytes constituted approximately 73-74% of the lymphocyte population (p = 0.319), while NK cells and B lymphocytes remained stable at approximately 12% and 9%, respectively (p = 0.571 and p = 0.526, respectively).

**Table 2:** Peripheral blood lymphocyte subset distribution in patients with silicone breast implants. SD: standard deviation; CD: cluster of differentiation; NK: natural killer; CTL: cytotoxic T lymphocytes; Treghigh: T regulatory high

Lymphocyte subpopulations	Mean value (%) ± SD			P-value
	Preoperatively	3 months postoperatively	12 months postoperatively	
CD3+ (T cells)	74.12 ± 5.41	73.52 ± 4.94	72.83 ± 4.99	0.319
CD3-/CD19+ (B cells)	8.85 ± 2.95	9.24 ± 2.78	8.96 ± 3.47	0.526
CD3-/CD16+/CD56+ (NK cells)	11.87 ± 5.77	13.08 ± 6.07	12.98 ± 5.45	0.571
CD3+/CD4+ (T-helper cells)	42.25 ± 8.19	40.91 ± 8.31	40.23 ± 8.29	0.189
CD3+/CD8+ (CTLs)	31.54 ± 6.77	31.21 ± 8.83	31.84 ± 7.17	0.883
CD4+/CD25high (Treghigh cells)	4.50 ± 1.15	4.87 ± 1.18	4.78 ± 2.04	0.457

Among the CD3+ T-lymphocyte subsets, T-helper cells (CD3+/CD4+) were the predominant population, followed by cytotoxic T lymphocytes (CTLs) (CD3+/CD8+, CTLs). T-helper cells accounted for 42.25% ± 8.19% of lymphocytes preoperatively, with a slight, non-significant decline to 40.91% ± 8.31% at three months and 40.23% ± 8.29% at 12 months postoperatively (p = 0.189). CTLs remained steady, with mean percentages of approximately 31-32% across all time points (p = 0.883). Additionally, the population of T-regulatory (Treg) cells (CD4+/CD25high, Treghigh cells) showed no significant temporal changes, with proportions hovering around 4.5-4.9% (p = 0.457).

**Figure 1:**
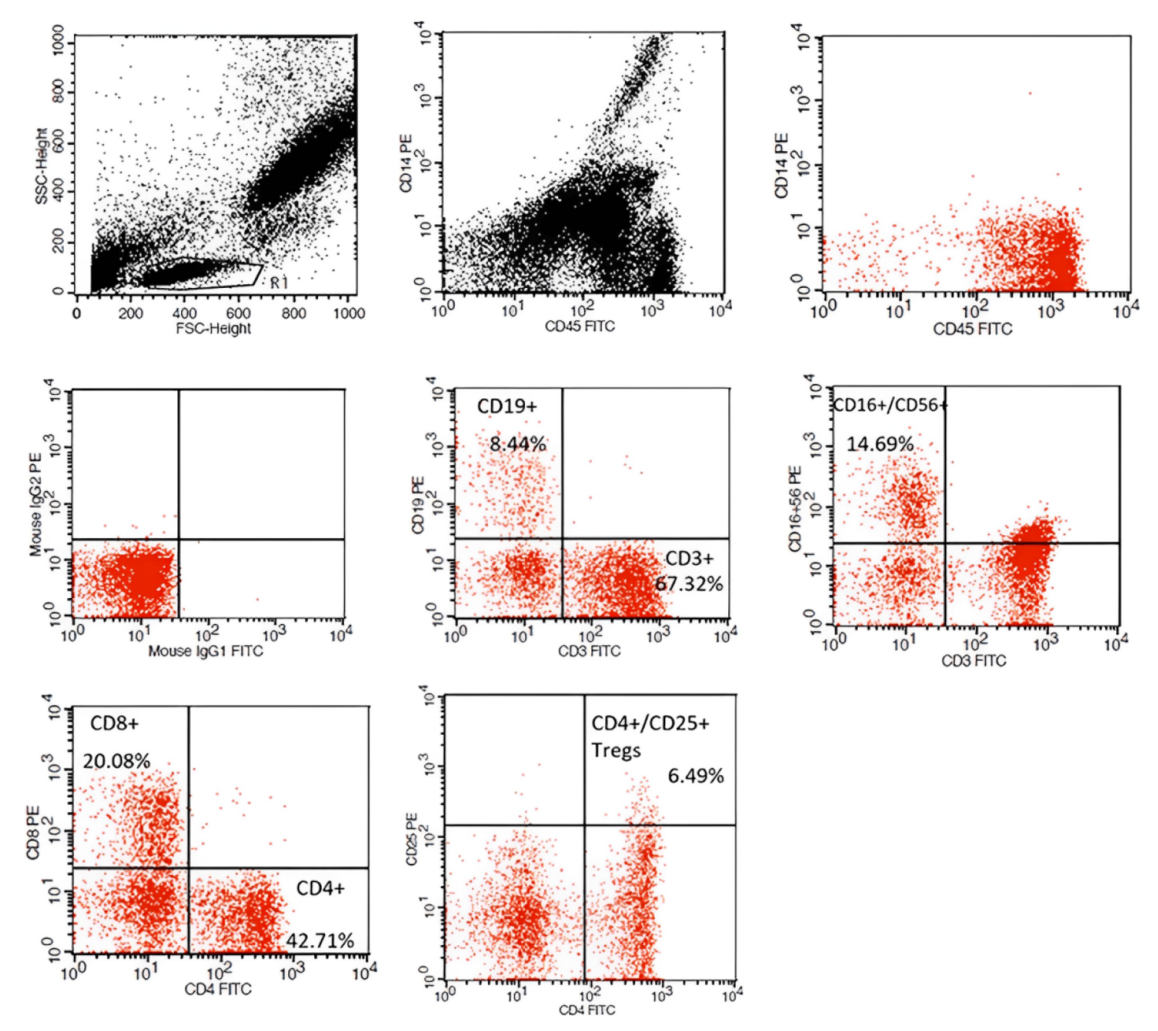
Flow cytometric gating strategy and contour plots illustrating lymphocyte subset analysis. The initial gating strategy excluded doublets and debris using FSC and SSC parameters, followed by gating on CD45+ leukocytes. Lymphocytes were subsequently identified within the FSC/SSC low population. Subsets of lymphocytes were identified as follows: CD3+ T cells, CD4+ helper T cells, CD8+ cytotoxic T cells, CD19+ B cells, CD16/CD56+ natural killer cells, and CD25+ activated T cells. FSC: forward scatter; SSC: side scatter; CD: cluster of differentiation; PE: phycoerythrin; FITC: fluorescein isothiocynate; Ig: immunoglobulin; Tregs: T regulatory cells

The flow cytometric gating strategy is illustrated in Figure 1, where doublets and debris were excluded using forward scatter (FSC) and side scatter (SSC) parameters, and leukocytes were gated based on CD45 positivity. Lymphocytes were further identified within the FSC/SSC low population, and distinct subsets were delineated based on marker expression. Contour plots provide representative data for the identification of T cells (CD3+), T-helper cells (CD4+), CTLs (CD8+), B cells (CD19+), NK cells (CD16+/CD56+), and activated T cells (CD25+).

Overall, a stable immunophenotypic profile of peripheral blood lymphocytes in patients undergoing silicone breast implant procedures was found. The absence of statistically significant changes in white blood cell or lymphocyte subset distributions across the preoperative and postoperative periods suggests that these surgical interventions do not elicit detectable alterations in systemic immune cell populations.

## Discussion

The impact of surgery on a patient’s immunologic profile could be detrimental and can induce several complications. During the immediate postoperative period, there is an immune activation at the site of injury, which induces a systemic anti-inflammatory response that in turn causes suppression of the cellular immunity. Postsurgical and posttraumatic immune suppression has been reported in different studies, including the increased percentage of neutrophils, decreased lymphocyte numbers, and reduced expression of human leukocyte antigen-DR (major histocompatibility complex class II) by circulating monocytes and lymphocytes [11-13].

The implantation of biomaterials, such as silicone medical devices can induce several local reactions, including acute and/or chronic inflammation, granulation tissue development, foreign body formation, and fibrous capsule formation around the implant [9]. Particularly, the biomaterial surface properties can play an important role in modulating foreign body reaction, and therefore, the safety, biocompatibility, and function of the device. Although silicone was originally regarded as being inert in the human body, its polymeric and hydrophobic characteristics and the presence of electrostatic charges and organic side groups render silicone a potentially ideal immunogen [14]. Several studies have reported the causal association between medical silicone materials and the development of various complications such as inflammatory reactions, rheumatic diseases, and lymphadenopathy, which may involve the activation of the immune system [15-17].

The tissue response to silicone implants is characterized by the formation of a fibrous capsule and, eventually, the accumulation of reactive and exudative fluid around the implant. Currently, there is evidence that liquid silicone droplets are surrounded by macrophages, foreign body giant cells, and other inflammatory cells within the capsular tissue in patients with capsular contracture after cosmetic breast augmentation [18]. Ojo-Amaize et al. reported the presence of elevated serum silicon levels in women with breast implants who had chronic fatigue, musculoskeletal symptoms (fibromyalgia, joint pain, muscle cramps, and arthritis), and skin disorders [19]. The evidence of local and systemic detection of silicone particles suggests that silicone may itself have antigenic properties, causing the activation of a humoral-mediated immunologic response. Different studies have identified several types of antibodies (antinuclear antibodies, anticollagen antibodies, anticardiolipin antibodies IgG and IgM, rheumatoid factor, anti-Ro, and anti-La) against different self-antigens in patients with silicone implants [20-23]. Increased levels of antibodies to silicone elastomers (antisilicone antibodies) have also been reported in the serum and capsular tissue of patients with tissue expanders or silicone breast implants, especially after leakage or frank rupture [24,25]. However, the presence of these antisilicone antibodies has little or no clinical importance, and further long-term studies are needed.

The ability of silicone breast implants to induce a specific systemic immune reaction versus a nonspecific local inflammatory response remains controversial. In our study, we demonstrated that there is no statistically significant difference in the temporal distribution of the peripheral blood leukocyte subsets (polymorphonuclear cells, monocytes, and lymphocytes). Interestingly, lymphocytes showed a slight increase and T-helper lymphocytes a slight decline postoperatively, with no evidence of statistically significant shifts. Therefore, we hypothesize that during the period of our study, there was no evidence of systematic immune reaction to the breast implants. Nair et al. reported that silicone nanoparticles up to 100 μg/mL did not influence the production and gene expression of tumor necrosis factor-alpha, interleukin-6, and interferon-gamma by PBMCs [26]. Similarly, Vanni et al. showed that prepectoral breast reconstruction does not affect early immunological response [27]. However, it has been demonstrated that in co-cultures, lymphocytes enhanced macrophage adhesion and fusion to the biomaterial surface, while the presence of monocytes increased the activity of co-cultured lymphocytes [28,29].

Despite the widespread use of silicone implants, there have been few systematic studies on the immunophenotyping analysis of the peripheral blood lymphocyte subpopulations in patients with silicone breast implants. With the use of flow cytometry, we demonstrated that most of the peripheral lymphocytes consisted of T lymphocytes (CD3+) either during the preoperative period or three months and 12 months postoperatively, followed by the NK cells (CD16+/CD56+) and B lymphocytes (CD19+). However, no statistically significant differences in the distribution of peripheral blood lymphocytes could be detected in patients with silicone breast implants. Smalley et al. reported an immunologic stimulation of T lymphocytes by silica after the use of silicone mammary implants [30], while Vojdani et al. found a significantly reduced ability by NK cells to kill tumor target cells in patients with silicone implants compared to healthy individuals [31].

In our study, the peripheral T lymphocytes were predominantly T-helper lymphocytes (CD3+/CD4+), followed by CTLs (CD3+/CD8+, CTLs). Similarly, Prantl et al. reported the predominance of T-helper cells in the serum of patients with capsular contracture after silicone-based breast augmentation; however, there was no statistically significant difference in the distribution of peripheral blood lymphocytes in comparison with healthy controls, and there was no evidence of systemic proinflammatory effects of silicone breast implants [32]. However, Katzin et al. demonstrated that capsular tissue-associated T lymphocytes showed an increased expression of CD29 and a decrease in the expression of CD45RO by CD4+ cells (phenotype, CD3+/CD4+/CD29+/CD45RO-) in comparison with peripheral blood T lymphocytes in patients with silicone breast implants [33]. Moreover, Ojo-Amaize et al. reported an abnormal CD4+ cell-proliferative response in women with silicone gel breast implants after exposure to silicon dioxide (silica), silicon, or silicone gel [19].

In addition, we determined the distribution of a specific T-cell subpopulation, namely, Treg cells (CD4+/CD25+/Foxp3+, Treg cells). Naturally occurring Treg cells constitute 5-10% of peripheral CD4+ T cells in normal humans, but only the CD4+/CD25high cells (Treghigh cells), which constitute 2-3% of CD4+ T cells, are really regulator cells [34,35]. Their main function is to suppress the activation and effector functions of autoreactive T cells and control the immunologic tolerance to self-antigens and non-self-antigens. We found that the expression of Treghigh cells remained invariable up to one year after surgery in patients with silicone breast implants. To our knowledge, this is the first study on Treg cell percentage in the peripheral blood of patients with silicone implants who did not demonstrate any immunologic-related symptoms. Wolfram et al. reported an increase in the percentage and suppressive activity of Treghigh cells (CD4+/CD25high/Foxp3+/CD127-) in the capsular tissue compared with the periphery in patients with peri-silicone implant capsular fibrosis, while numbers of intracapsular Treghigh cells were inversely proportional to the clinical degree of capsular fibrosis [36].

Breast implants are considered safe and inert biomaterials. Nevertheless, there is evidence that a type of anaplastic large cell lymphoma (ALCL) is associated with breast implants [37,38]. ALCL is a rare T-cell lymphoma involving the capsular tissue and/or effusions associated with breast implants. Clinical findings include late-onset seroma, swelling, a palpable breast mass, pain, capsular contracture, and axillary lymphadenopathy, where the long-term outcomes of those patients appear to be unusually benign compared to other systemic ALCLs [39]. Flow cytometry can aid in the diagnostic approach for ALCL through the examination of tissue samples, effusions, and peripheral blood samples in patients with signs of local inflammation after implant-based breast augmentation as tumor cells usually express the phenotype CD3+/CD4+/CD30+/ALK- and CD3+/CD8+/CD30+/ALK- [40-42].

An important strength of this study is that the same patients who underwent silicone breast implantation were used as the control group as flow cytometric analysis included preoperative peripheral blood sampling. Then, we performed blood sampling during the three-month follow-up period, where postoperative signs and symptoms (mainly swelling, ecchymosis, and pain) completely subsided, which could potentially affect the status of the immune system as well as the validity of our study. Interestingly, different studies have investigated possible immune alterations in the surgical patient, proceeding to the collection of blood samples within a month from the operation and analyzing the leukocyte subtypes with flow cytometry, demonstrating different mechanisms of postoperative immunosuppression [13,43,44]. Finally, patients provided blood samples at 12-month follow-up, where the healing process has been completed [45]. This specific timepoint is usually the last follow-up of the patients who underwent breast augmentation as compliance with the surgeon’s recommendations for follow-up visits declined after the first postoperative year [46,47].

There are several inherent limitations of our study, regarding mainly the small number of patients included in the statistical analysis, and the retrospective analysis of the data, as many patients were lost during the follow-up period. Moreover, we acknowledge that our study examines a specific and specialized aspect of the immune response rather than a comprehensive immunologic evaluation. While our findings suggest no significant systemic immune reaction to silicone implants within the study period, future research should incorporate broader immune parameters, including cytokine profiles and functional immune assays, to provide a more complete understanding of the immunologic effects of silicone implants. The lack of diversity in surgeons and implant types could limit generalizability. The last peripheral blood sample was obtained 12 months postoperatively, which makes our results an indicator of the early immune response to silicone implants. Therefore, further long-term follow-up is needed to assess with higher sensitivity the immunologic activity of those patients.

## Conclusions

We provide evidence that silicone breast implants are inert biomaterials that can be used safely for aesthetic and reconstructive purposes in breast surgery. However, further scientific research with a larger number of patients and long-term follow-up is needed to study more accurately the systemic immune response to breast implants. Flow cytometry enabled the assessment of immunophenotypic expression of peripheral lymphocytes in patients with silicone implants by tracking changes over time and found that no statistically significant changes were observed in lymphocyte distribution. Additional studies are needed to determine its role in evaluating the biocompatibility of silicone implants and its potential as a screening tool for immune responses in this clinical setting.
